# Octenidine’s Efficacy: A Matter of Interpretation or the Influence of Experimental Setups?

**DOI:** 10.3390/antibiotics11111665

**Published:** 2022-11-19

**Authors:** Djenana Vejzovic, Azra Iftic, Ayse Ön, Enrico F. Semeraro, Nermina Malanovic

**Affiliations:** 1 Institute of Molecular Biosciences, University of Graz, 8010 Graz, Austria; 2 Field of Excellence BioHealth, University of Graz, 8010 Graz, Austria; 3 Bio TechMed Graz, 8010 Graz, Austria

**Keywords:** octenidine, reduced susceptibility, resistance, adaption, tolerance, mechanism of action, multidrug resistance, inoculum effect, *E. coli*, cell density, binding to bacteria, MIC, lipid mutants, QUANTOM^TM^, Bioscreen

## Abstract

With its broad antimicrobial spectrum and non-specific mode of action via membrane disruption, any resistance to octenidine (OCT) seems unlikely and has not been observed in clinical settings so far. In this study, we aimed to investigate the efficacy of OCT against *Escherichia coli* and mutants lacking specific lipid head groups which, due to altered membrane properties, might be the root cause for resistance development of membrane-active compounds. Furthermore, we aimed to test its efficacy under different experimental conditions including different solvents for OCT, bacterial concentration and methods for analysis. Our primary goal was to estimate how many OCT molecules are needed to kill one bacterium. We performed susceptibility assays by observing bacterial growth behavior, using a Bioscreen in an analogous manner for every condition. The growth curves were recorded for 20 h at 420–580 nm in presence of different OCT concentrations and were used to assess the inhibitory concentrations (IC_100%_) for OCT. Bacterial concentrations given in cell numbers were determined, followed by Bioscreen measurement by manual colony counting on agar plates and QUANTOM^TM^ cell staining. This indicated a significant variance between both methods, which influenced IC_100%_ of OCT, especially when used at low doses. The binding capacity of OCT to *E. coli* was investigated by measuring UV-absorbance of OCT exposed to bacteria and a common thermodynamic framework based on Bioscreen measurements. Results showed that OCT’s antimicrobial activity in *E. coli* is not affected by changes at the membrane level but strongly dependent on experimental settings in respect to solvents and applied bacterial counts. More OCT was required when the active was dissolved in phosphate or Hepes buffers instead of water and when higher bacterial concentration was used. Furthermore, binding studies revealed that 10^7^–10^8^ OCT molecules bind to bacteria, which is necessary for the saturation of the bacterial surface to initiate the killing cascade. Our results clearly demonstrate that in vitro data, depending on the applied materials and the methods for determination of IC_100%_, can easily be misinterpreted as reduced bacterial susceptibility towards OCT.

## 1. Introduction

In contrast to antibiotics, antiseptics are currently thought to occupy a unique position in causing no antimicrobial resistance due to their unspecific mode of action. However, this assumption has been challenged in recent years. In particular, the widely described clinical manifestations of resistance (including even cross-resistance to antibiotics) to chlorhexidine (CHG), one of the most important antiseptic agents worldwide, has raised serious concerns among healthcare professionals about its use for preventive measures in infection control. Therefore, concerns about the existence of such antimicrobial resistance to octenidine (OCT), which has been clinically used for more than 30 years in Europe, and more recently, in Australia and Asian countries for skin, mucous membrane and wound antisepsis as well as microbial decolonization of patients, are understandable.

OCT is a small, synthetic pyridine derivative N,N′-(1,10 decanediyldi-1[4H]-pyridinyl-4-ylidene)-bis-(1- octanamine) dihydrochloride] with a broad spectrum of antimicrobial activity against (multidrug-resistant) bacteria and fungi [1,2,3,4,5,6,7,8]. Several studies showed very similar values for minimal inhibitory concentration (MIC) and minimal bacterial concentration (MBC) among different species [2,9,10]. Surprisingly, Shepherd et al. [11] reported in 2018 the adaptation of clinical isolates of *Pseudomonas aeruginosa* to OCT, and Wand et al. [12] described an efflux pump SmvA responsible for OCT tolerance in *Klebsiella pneumoniae*. The same group demonstrated in a recent publication [13] a mechanism by which *P. aeruginosa* adapts to OCT. In particular, OCT tolerance was associated with the respective efflux pump and remodeling of the membrane due to mutations in phosphatidylserine synthase *pssA* and phosphatidylglycerolphosphate synthase *pgsA,* thereby affecting membrane composition and order. However, discrepancies arose when Conceicao et al. showed for Gram-positive bacteria, that clinical MRSA isolates harboring genes encoding multidrug resistance efflux pumps mediating antimicrobial resistance remained completely sensitive to low concentrations (0.001%) of OCT [5]. These results are supported by findings from Htun et al., who examined the resistance pattern of 878 MRSA isolates after decolonisation of patients by using CHG or OCT, respectively [14]. Antiseptic exposures were associated with the carriage of *qac* genes, however, quite in contrast to CHG, the presence of efflux pumps had no effect on the susceptibility of the respective strains to OCT. Moreover, a very recent publication further strengthens the position of OCT with regard to undesired resistance and even cross-resistance to CHG or the last-resort antibiotic colistin in different Enterobacterial species even under high selective pressure in vitro [15]. In addition, our actual data on the mode of action of OCT on Gram-negative bacteria using *Escherichia coli* as a model organism [10] and Gram-positive species using *Enterococcus hirae* and *Bacillus subtilis* [9] underpin membrane disruption as a clear non-specific mechanism. The insertion of OCT into the membrane results in a chaotic lipid arrangement that is not based on specific interaction with the lipid head groups, but strongly affects fatty acyl tails [10]. We also showed that OCT disrupts membranes composed of any bacterial lipid and further, *B. subtilis* mutants defective in production of various phospholipids, including *pssA* and *pgsA*, are still susceptible to OCT to a similar extent as the wildtype [9].

In the present study, we expand on our previous work performed with Gram-negative bacteria in order to: (1) look more comprehensively at the influence of charged lipids in bacterial membranes on the activity of OCT and to (2) further clarify discrepancies in published studies regarding the observed reduction of bacterial susceptibility to OCT. Gram-negative bacteria are surrounded by two membranes, an outer membrane containing lipopolysaccharide (LPS) and a thin cytoplasmic membrane, and are therefore complex in architecture. Knowing that their membrane composition significantly differs from Gram-positive bacteria [16], we evaluated in the first step OCT´s antimicrobial activity with respect to membrane remodeling using diverse mutants of *E. coli* that are unable to produce major membrane lipids: LPS, phosphatidylglycerol (PG) and/or cardiolipin (CL). Since previous data showed that media composition and plate material can influence the antiseptic activity of OCT in vitro [17], we further evaluated if: (1) solubility of OCT in various buffer systems, as well as bacterial numbers relative to a given concentration of OCT have an impact on its activity, and (2) different established methods to measure bacterial concentration influence the outcome of MIC determination. Finally, we investigated the number of OCT molecules per cell required for bacterial killing.

## 2. Results

### 2.1. OCT’s Antimicrobial Activity Is Not Altered in E. coli Mutants Deficient in LPS, PG and CL

We recently reported for the Gram-positive bacterium *B. subtilis* that OCT induced membrane disorder independently of the magnitude of surface charge or presence of different lipid head groups. Mutants lacking specific head groups were as sensitive to OCT as the wildtype [9,10]. Here, we determined OCT’s antimicrobial activity in mutants of *E. coli* deficient in the production of major anionic phospholipids CL and PG (BKT12 and BKT29; Table 1). Experiments were also performed in the *E. coli* wildtype (ATCC25992) that produces full length LPS, in K12 derivatives (MG1655, BW25113 and d21) lacking the O-Antigen of LPS, in addition to the *E. coli* mutant d21f2 that is unable to produce O-Antigen and the inner and outer core of LPS. OCT exhibited the same MIC value for all tested strains, indicating a non-selective mechanism, irrespective of membrane lipids and possible escape mutations due to lipid remodelling in the membrane of a Gram-negative bacteria. In the following, all experiments were performed with the *E. coli* wildtype ATCC25992 strain. Furthermore, antimicrobial activity was expressed as IC_100%_, which represents the total inhibitory concentration of OCT resulting in no visible growth at bacterial concentrations higher than those defined for standard MIC determination at 1 × 10^6^ CFU/mL.

### 2.2. OCT’s Antimicrobial Activity Is Influenced by the Buffer System and Bacterial Concentration in Relation to a Given Dose of Antiseptic

**Figure 1:**
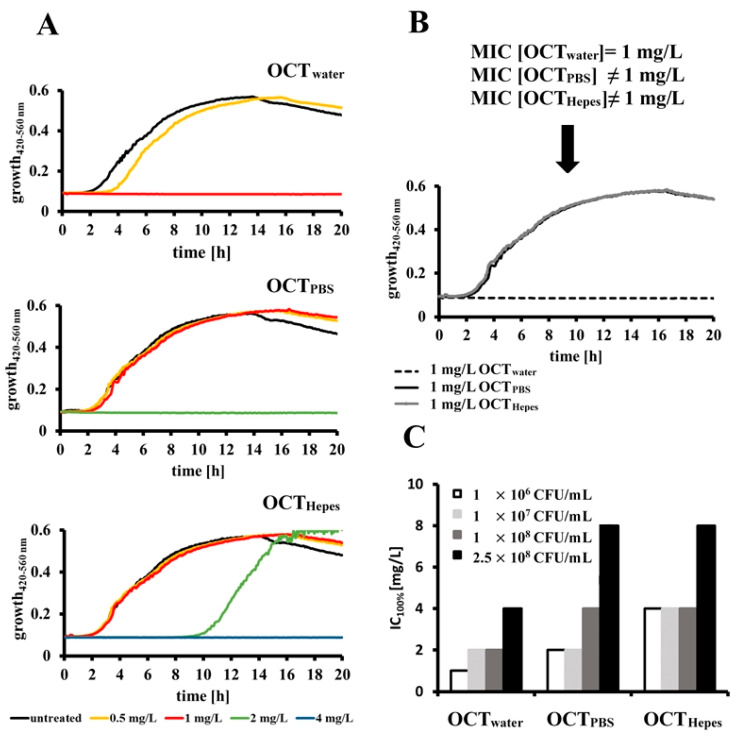
**Inhibition of *E. coli* growth is strongly influenced by OCT solvent and bacterial cell counts.** (**A**) Growth curves of 1 × 10^6^ CFU/mL *E. coli* wildtype recorded in presence and absence of the indicated OCT concentrations over 20 h by Bioscreen at 420–560 nm. OCT was dissolved in water (upper panel), PBS (middle panel) or Hepes buffer (lower panel) before being added to the initial bacterial cell suspension. (**B**) Growth of *E. coli* treated with 1 mg/L OCT dissolved in water (dotted line), PBS (black line) and Hepes buffer (grey line). MIC values represent the lowest concentration of OCT that prevented the growth of 1 × 10^6^ *E. coli*. (**C**) Total inhibitory concentration (IC_100%_) for OCT dissolved in the indicated buffer systems against *E. coli* of different concentrations ranging from 1 × 10^6^–2.5 × 10^8^ CFU/mL, as determined by Bioscreen at 420–560 nm. All results are representative data of at least three independent experiments.

To the best of our knowledge, it is unknown whether the buffer system in which OCT is dissolved has the potential to influence the activity profile of OCT. Therefore, we dissolved the antiseptic in the most frequently used fluids in bacterial in vitro research, namely water (OCT_water_), phosphate-buffered saline (OCT_PBS_) and Hepes buffer (OCT_Hepes_) and assessed its antimicrobial efficacy against wildtype *E. coli*. Growth curves of 1 × 10^6^ CFU/mL *E. coli* were recorded in the absence and presence of OCT ranging from 0.1 to 16 mg/L (Figure 1A; for clarity only the curves using 0.5–4 mg/L OCT are shown). OCT_water_ completely abolished growth of *E. coli* at 1 mg/L, whereas 2 mg/L of OCT_PBS_ and 4 mg/L of OCT_Hepes_ were required to observe the same inhibition. Figure 1B clearly shows that *E. coli* cells exposed to 1 mg/L OCT exhibit normal growth behavior when dissolved in both buffering systems, while in contrast, no growth was observed for OCT_water_.

In the next step, we investigated whether the OCT solvent has an impact on its antimicrobial activity in the presence of several bacterial concentrations (Figure 1C). Interestingly, we found that higher bacterial cell counts required more OCT molecules to completely inhibit microbial growth. With increasing bacterial concentrations from 1 × 10^6^ to 2.5 × 10^8^ CFU/mL, IC_100%_ for OCT_water_ changed from 1 mg/L up to 4 mg/L, for OCT_PBS_ from 2 mg/L to 8 mg/L and for OCT_Hepes_ from 4 mg/L to 8 mg/L, respectively. These results show that calculated IC_100%_ values differ significantly between the tested OCT solutions and are even further influenced by the initial bacterial cell count. Thus, especially in the presence of very low OCT concentrations, some cells may not contact a sufficient number of antiseptic molecules and consequently, bacterial growth could be misinterpreted as reduced susceptibility or tolerance.

### 2.3. Determined IC_100%_ Values Are Influenced by Different Methods for Estimating Bacterial Cell Counts

The so called “inoculum-effect”, described first for antibiotics, e.g., quinolones or penicillin [30], is a well-known phenomenon that affects the activity profile of antimicrobial peptides by changing the concentration of bacterial cell density [31,32,33]. Our previous studies [9,10] indicated that a specific number of OCT molecules per bacteria might be necessary to induce the killing cascade and consequently disrupt the membrane. As shown in Figure 1C, an increase in antimicrobial activity of OCT_water_ (from 1/2/2 to 4 mg/L) correlated with elevated bacterial concentrations (1 × 10^6^/1 × 10^7^/1 × 10^8^/2.5 × 10^8^ CFU/mL). To follow up on these observations, we first speculated about the accuracy of our standard method of determining *E. coli* cell count, which is routinely measured by the optical density (OD) of the cell suspension and recalculated to a CFU/mL value.

We compared results from wildtype *E. coli* by using the most common methods, which are: (1) absorbance/OD followed by direct recalculation of OD_600_ = 1 to 8 × 10^8^ CFU/mL, (2) manual counting of colonies on agar plates after incubation for 24 h at 37 °C and (3) measurement of total cells according to QUANTOM^TM^ methodology. In fact, results from the 3 methods differ in our laboratory by 25–50% (Table 2).

**Figure 2:**
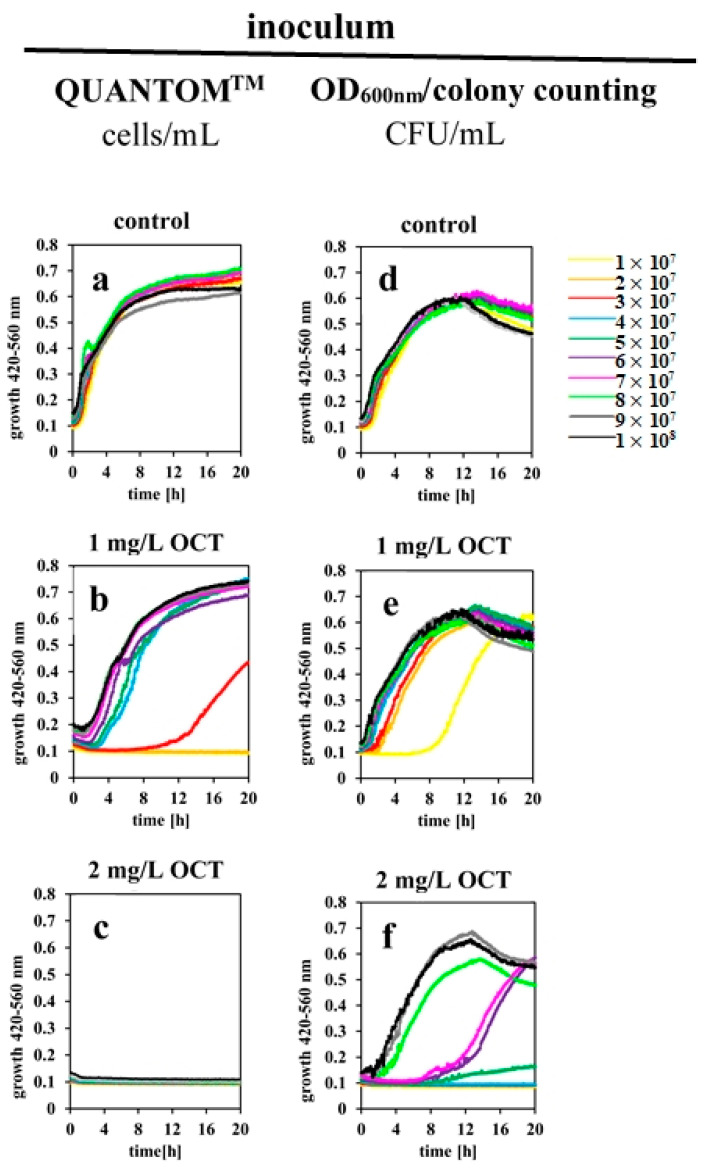
**Inhibition of *E. coli* wildtype growth is strongly influenced by OCT_water_ concentration and initial bacterial load.** Growth curves of inoculum of 1 × 10^7^–1 × 10^8^ cells/mL or CFU/mL as measured directly by QUANTOM^TM^ technology (**a**–**c**) or optical density following the recalculation of CFU/mL by colony counting on agar plates (**d**–**f**), respectively. Bacterial growth in the absence (**a**,**d**) and presence of indicated OCT concentrations (**b**,**c**,**e**,**f**) over 20 h as recorded by Bioscreen at 420–560 nm. The results are representative data of at least three independent experiments.

Based on these findings, we conducted a series of experiments using manual colony counting on agar plates as well as QUANTOM^TM^ total cell staining to investigate the antimicrobial activity of OCT_water_ in the presence of different wildtype *E. coli* cell counts. We performed microdilution assays with bacterial concentrations ranging from 1 × 10^7^ to 1 × 10^8^ CFU/mL (or cells/mL), respectively, with or without antiseptic at constant OCT concentrations of 0.5, 1 and 2 mg/L. When the inoculum concentration was determined by QUANTOM^TM^ technology (Figure 2a–c), a slight delay in growth of *E. coli* was observed in the presence of 0.5 mg/L OCT (data not shown) which became more significant at 1 mg/L OCT. Here, bacterial growth was close to the control (at ≥4 × 10^7^ cells/mL), shifted drastically at 3 × 10^7^ cells/mL and was completely abolished when 2 × 10^7^ cells/mL or less bacteria were used (Figure 2b). At 2 mg/L OCT no bacterial growth was observed (Figure 2c). However, when the inoculum concentration was estimated according to the colony counting approach (Figure 2d–f), growth curves recorded in presence of 1 mg/L OCT also showed a substantial impact but never caused complete growth inhibition (Figure 2e). This was achieved first at an initial 4 × 10^7^ CFU/mL in the presence of 2 mg/L OCT (Figure 2f). Interestingly, when 5–7 × 10^7^ CFU/mL were used in this setting, very few bacteria survived, resulting in long delay of growth. In conclusion, our data indicate a significant variance in measured IC_100%_ values, which are strongly dependent on the accuracy of the underlying method for determining cell count, especially at low concentrations of OCT.

### 2.4. Low Numbers of OCT Molecules Are Taken Up by Bacteria

**Figure 3:**
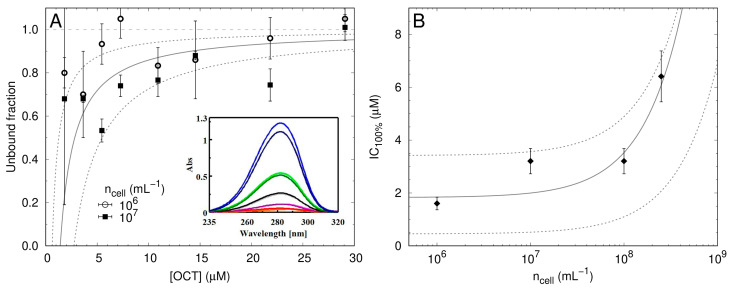
Estimating the number of OCT molecules binding to an *E. coli* cell. (**A**). The unbound fraction of OCT_water_ as estimated by 281 nm after exposure to 1 × 10^6^ and 1 × 10^7^ cells/mL dissolved in PBS. OCT was applied at concentrations in the range where it exerts antimicrobial activity against *E. coli* and above the IC_100%_ (IC_100%_ for 1 × 10^6^ CFU/mL and 1 × 10^7^ CFU/mL in PBS buffer is 2 mg/L). The insert shows absorbance spectra of OCT at 281 nm. The lowest spectra correspond to the lowest OCT concentrations and their absorbance intensity increased with increasing OCT concentrations. In the presence of bacterial cells, the slightly lower absorbance in all spectra was observed indicating association of OCT with bacteria. Measurements were performed at least three times. To obtain the number of molecules per cell, the bound (partitioned) fraction is calculated by subtracting the unbound (free) fraction from the overall OCT concentration. In this case, the bound fraction is proportional to bacterial concentration, and results in an estimate of 1–2 × 10^8^ OCT molecules per single cell at IC_100%_. (**B**) IC_100%_ values as a function of cell concentration. MIC or IC_100%_ corresponding to OCT´s concentration where no bacterial growth was observed, were calculated from growth curves recorded in MHB for OCT_water_ in presence of different bacterial concentrations (1 × 10^6^, 1 × 10^7^, 1 × 10^8^ and 2.5 × 10^8^ CFU/mL). To obtain the number of molecules per cell, the overall OCT concentration is divided in bound and unbound fractions. Free OCT is independent of cell concentration and is revealed at low bacterial presence (about less than 2 mM [1.2 mg/L]). The steep increase of IC_100%_ instead is due to the bound OCT fraction. It is linear with cell concentration and enables the extraction of OCT molecules bound on a single cell (it is around 1 × 10^7^). Solid lines are the best data fits, and dashed lines enclose the area accounting for the level of confidence.

In order to estimate the maximum number of OCT molecules which are able to bind to a single bacterium, we performed two calculations based on published procedures, as described more precisely in the Materials and Methods (Section 4.4). Both approaches enable calculation of how many OCT molecules bind to a single cell (Figure 3). As depicted in Figure 3A, the first calculation results from measuring the absorbance of OCT at 281 nm after incubation with bacteria of defined concentration (insert), showed an estimated 1–2 × 10^8^ OCT molecules are required per cell to achieve IC_100%_. The second calculation (Figure 3B) is derived by modelling IC_100%_ values obtained from microdilution assays [33] and resulted in approximately 1 × 10^7^ OCT molecules per cell. Based on these calculations, it appears that OCT molecules remain associated with the cell after triggering cell death. The difference between these two estimations is due to the applied methodology: the first approach “counts” the number of bound OCT molecules per cell after 5 min of incubation, whereas the second approach gives the number of bound OCT per cell that cause bacterial growth inhibition.

Interestingly, in both scenarios a larger number of unbound antiseptic molecules is estimated and only a smaller portion of OCT appears to be taken up by bacterial cells. It is important to note that this observation strongly depends on both cell count and OCT concentration. It is also important to note that overestimating the cell concentration will result in an underestimation of the number of OCT per cell, and vice versa. Figure 3A, for example, shows that in the IC_100%_ range (about less than 2 mg/L [3.2 μM] at 1 × 10^6^ CFU/mL and 1 × 10^7^ CFU/mL) this portion increases up to 30–50% of total applied OCT, and diminishes only when an excess of OCT, above the IC_100%_ value, is used. Since in these conditions, most of the OCT remains free in solution at IC_100%_ concentration, it can be assumed that a significant degree of OCT saturation outside the cells is necessary to kill the bacteria.

## 3. Discussion

Antimicrobial resistance is rising to dangerous levels worldwide, threatening treatment options for successful patient outcomes. In that context, antiseptics constitute a suitable tool, not only in the field of infection prevention by reducing the transmission of (multi)drug resistant microorganisms in healthcare facilities, but also for local therapy. However, for some widely used antiseptics, reduced bacterial susceptibility and even (cross-)resistance to antibiotics have been reported recently.

Generally, the accurate dosage and application interval of antimicrobial agents plays an important role, as the formation of resistance is closely related to their administration at sublethal doses and/or prolonged timeframes. Using concentrations inadequate for microbial killing may lead to the survival of mutants and the induction of antimicrobial resistance [34]. Parameters such as MIC or MBC are used to describe antimicrobial susceptibility in vitro, and the obtained values can, if reliably assessed, help quantitatively describe the efficacy of a specific antimicrobial. However, the credibility of these values is dependent on multiple factors, including a determination method and the interpretation of results regarding clinical relevance [35].

Recent in vitro studies reported an increased tolerance to the clinically widely used antiseptic OCT in Gram-negative Enterobacterial species that is related to (i) efflux pump [12,13]; (ii) membrane remodeling by preventing the production of the anionic phospholipid phosphatidylserine (PS) and phosphatidylglycerol (PG) [13] and (iii) sequestering of cationic OCT molecules by components of the growth media or assay plates [17]. Of note, in all these experiments, only extremely low concentrations of OCT were used, far away from those used for clinical application (0.05–0.1%). Other studies excluded efflux pumps as a possible resistance mechanism for OCT in Gram-positive bacteria [5,14] as well in Enterobacterial species [15]. Furthermore, recent studies from our group [9,10], which indicated a non-selective phospholipid targeting of OCT, challenge the assumption by Bock et al. [13] that lipid remodeling may affect OCT´s activity to such an extent that it might induce bacterial resistance. We showed that depletion of any lipid identified in *B. subtilis* cell envelope as lipoteichoic acid, glucolipid, lysophosphatidylglycerol, cardiolipin, phosphatidylethanolamine or PS did not strongly affect OCT´s antimicrobial activity. At a molecular level, we also showed that OCT was most effective in disrupting membranes composed of neutral phospholipids, but it was also potent in breaking anionic membranes or membranes with different properties as found in mixed phospholipid membranes [9]. We concluded that OCT favours hydrophobic over electrostatic interactions, and this hypothesis is further strongly supported by the data presented in this manuscript: mutants of *E. coli* defective in the production of major anionic membrane lipids, truncated LPS or CL and PG are not able to impair OCT´s activity at all (Table 1). Cationic compounds, such as the antimicrobial peptide PMAP23, generally show increased activity towards mutants lacking anionic lipids [36]. Therefore, the most probable explanation is that anionic lipids preclude their association to and perturbation of the cytoplasmic membrane [37]. Nevertheless, in the case of OCT, electrostatic interactions seem not to be the driving force for its mode of action, as *E. coli* BKT29 mutant lacking both anionic phospholipids, PG and CL, is equally susceptible to OCT as the wildtype strain (Table 1). For that reason, in respect to lipid depletions and in relation to membrane remodelling that might occur upon defective lipid metabolism in bacteria [37,38], it is less conceivable that OCT´s activity might be affected upon membrane remodeling than has been reported previously [13]. Taken together, our results clearly show OCT´s capability to kill bacteria of different membrane compositions and to disrupt membranes of different composition. It is worth mentioning in this context that cytoplasmic membranes of *E. coli* and other Enterobacterial species such as *P. aeruginosa* do not significantly differ in their phospholipid composition [16,38], and certain variations of membrane composition in respect of OCT´s action are less recognized.

Although standardized procedures for MIC and MBC assessment exist, mainly for antibiotics, in case of antiseptics and antimicrobial peptides, there are still some limitations that are encountered [16]. In particular, different growth media compositions and various methods for assay monitoring further complicate an accurate correlation of in vitro effects. As a result, data obtained from individual laboratories are often not compatible, especially when experiments are performed with amphipathic molecules like OCT, which can interact with many components of utilized research materials [39,40]. Indeed, an earlier publication demonstrated the importance of media composition and plate material on antiseptic activity in vitro [17]. Brill et al. [40] also noticed that a low uptake of OCT due to its binding to components of the growth media apparently led to a reduced killing rate. Altogether, these studies further highlight the need for optimized testing methods to obtain reliable and comparable results under laboratory conditions. Indeed, we have encountered and showed herein that under the very same experimental conditions, MIC values vary between 1 and 4 mg/L and even further increase when higher *E. coli* concentrations are used, depending on the respective buffer used to dissolve OCT (water, PBS or Hepes buffer; Figure 1). The critical micelle concentration of OCT in ultrapure water was reported at 3.79 mM (= 2 g/L = 0.2%) [41], which is about 1,000 times higher than its activity profile [4,10]. Interestingly, in preliminary tests we observed a strong potential of OCT to precipitate in different buffers, usually at concentrations above 0.01% (data not shown), at which large white floccules were visible to the eye. The same observation was made when 1% OCT stock solution in water was stored at 4 °C.

In that context, recent studies by Rzycki et al. on the simulation of artificial bacterial membranes composed of only phospholipids revealed that OCT first forms aggregates before it interacts with the membrane: the occupied area of OCT in the membrane requires ~0.5 nm^2^, which is similar to the area of a standard phospholipid (0.55–0.6 nm^2^) [42]. The authors assumed that an aggregation of OCT molecules is needed to reduce the entropy of the system. According to estimation derived from our binding studies, 1 × 10^7^ bound OCT per cell can cover the outermost bacterial surface entirely (i. e. ~1–2 OCT per nm^2^) and potentially exceed the number of LPS (~1–2 OCTs per LPS), located on a surface of a typical *E. coli* bacterium (assuming a cylinder of 1 µm diameter and 2 µm length). Indeed, after 5 min of incubation, we determined that the number of OCT molecule per cell was about 10 times the number required to saturate the bacterial surface and cause cell death. This phenomenon was already investigated in more detail by Stella´s group and others for different membrane-active antimicrobial peptides (AMPs) in *E. coli* as well as in artificial model membranes ([16,43,44,45]). These studies showed that a high membrane coverage is necessary for membrane perturbation. In any case, it was concluded that microbicidal mechanism in vitro can only be achieved when the membrane-active compound reaches the threshold concentration necessary to kill the bacteria. From this it is also plausible that the affinities to other components such as cell debris [36], or aggregation of OCT molecules affected by (salt) ions [42] might determine OCT´s local concentration on the membrane for bactericidal activity. In summary, our predictive model based on theoretical calculations describes the trend observed for the cell density dependence of MIC values for membrane-active compounds, mainly based on two assumptions: (1) a threshold of cell-bound OCT molecules is needed for bacterial killing, and (2) OCT association to bacteria is approximately described by a partition equilibrium. Most importantly, our results demonstrate that the OCT dosage plays a crucial role in controlling its use.

Following our assumption that a defined quantity of OCT molecules per single bacterial cell might be necessary for killing, we further provide important evidence that various standard methods for estimating bacterial cell counts result in different values of OCT’s inhibitory concentration (Figure 2). Importantly, the difference between those methods is not only in the range of a few cells, but it consists of 60 million cells/mL. Unfortunately, this is often not considered in experimental settings. As a matter of fact, this is of particular relevance when OCT is utilized at low concentrations. Our binding studies revealed that immediately after exposure of the cell to OCT at IC_100%_, only approximately a quarter of the total amount of OCT molecules appear to interact directly with bacteria. However, even this number (1 × 10^8^ OCT/cell) exceeds the total coverage of all lipid molecules (not only LPS) in one *E. coli* cell (4.5 × 10^7^ lipids/cell). This once again implies that, similar to studies by Stella et al. with AMPs [31,32,36,46], a threshold for OCT concentration is needed for membrane perturbation and hence, bacterial killing. It is therefore plausible that another 60 million bacterial cells/mL require more OCT molecules to exhibit the same bactericidal effect. The apparent error of 25 to 50% associated with two different measurements of cell counts can thus drastically influence results under laboratory conditions. This might depend on the bacterial concentration exposed to OCT, the sequestering of OCT molecules by other present compounds, or the influence of potential OCT aggregation as reported previously [9,10,17,41,42]. In this context, simulation of bacterial tolerance by in vitro assays, whereby a certain volume of bacterial culture without exact determination of bacterial concentration is reinoculated over several passages and exposed to antimicrobial agents, should be very critically questioned. Caution should be taken when interpreting those data to be the result of the development of resistance, tolerance or adaptation to an investigated compound. In general, measures in global healthcare have to be taken, since recent reports indicate growing resistances also for the last-resort antibiotics like carbapenems [47,48].

In summary, our results presented herein provide evidence that OCT´s antimicrobial activity in *E. coli* is not affected by changes at the membrane level. Mutants defective in production of major constituents of bacterial membranes, LPS, PG and CL are susceptible to OCT, as it is the wildtype. For its potent bactericidal activity, a high OCT coverage of the bacterial surface needs to be accompanied with a high proportion of OCT molecules in solution that do not directly bind to the cells. This important threshold concentration of OCT, necessary for initiating the killing cascade, might be further affected in vitro by different ions (as present in PBS and Hepes buffer), bacterial cell density or other reported factors such as the aggregation of OCT on the bacterial surface or binding to other components (e. g. growth media, plate material, cell debris). Consequently, bacterial growth in vitro, especially in the presence of very low concentrations of OCT, could be misinterpreted as tolerance or adaption. Altogether, in our view, due to its unspecific mode of action targeting critical parts of bacterial membranes, the development of resistance towards OCT is unlikely.

## 4. Materials and Methods

### 4.1. Preparation of Reagents

#### 4.1.1. Preparation of OCT Solutions

The stock solution (1% OCT dissolved in pure water; Schülke and Mayr GmbH, Austria) was further dissolved in either pure water, phosphate-buffered saline (PBS, 20 mM Na_2_HPO_4_/NaH_2_PO_4_, 130 mM NaCl, pH 7.4) or Hepes buffer (10mM Hepes, 140 mM NaCl, pH 7.4) to required concentrations.

#### 4.1.2. Preparation of Bacterial Cultures

Overnight cultures were made from freshly grown *E. coli* cells in Müller Hinton broth (MHB, Carlroth, Austria) under shaking at 37 °C. All strains used in the study are shown in Table 1. *E. coli* mutants d21, d21f2, BKT12 and BKT29 were kindly gifted by Lorenzo Stella and Maria Luisa Mangoni (University of Roma, Italy). The cells were further reinoculated in fresh MHB to OD_600 nm _= 0.05 and grown for 3.5–4 h to reach the mid-log phase. Optical density of the culture was then measured at 600 nm, and cells were washed two times using PBS and their concentration was determined as described in Section 4.2.

### 4.2. Estimation of Bacterial Cell Concentration

#### 4.2.1. CFU/mL by Literature Obtained Values

Colony forming units (CFU/mL) were recalculated from the absorbance measured by OD_600 nm,_ whereby according to the literature, an OD of 1 corresponds to 8 × 10^8^ CFU/mL [49].

#### 4.2.2. CFU/mL by Counting Colonies

Bacterial culture of desired optical density was plated on diagnostic agar plates and incubated overnight at 37 °C. CFU/mL was estimated by manual counting of the colonies of the respective culture.

#### 4.2.3. Cells/mL Using QUANTOM^™^ Total Cell Staining

The number of cells/mL was determined using the QUANTOM^™^ Total Cell Staining Kit (Biocat, Austria) according to the manufacturer´s instructions. The QUANTOM^™^ Total Cell Staining Dye is membrane permeable and fluoresces upon binding to nucleic acids in living and dead bacterial cells. The total cell number was obtained directly from microbial cell counter QUANTOM^™^(Biocat, Austria) allowing the counting of single cells.

### 4.3. Assessment of Bacterial Survival in the Presence of OCT by Micro Dilution Assay

Stock solutions of bacteria were made from washed mid-log *E. coli* cells in fresh MHB media to the required concentrations, ranging from 1 × 10^6^ to 2.5 × 10^8^ CFU/mL. The bacterial stock solutions are equal to term inoculum. 90 µL of bacterial culture was incubated with 10 µL OCT stock solution to final concentration of 0.1 mg/mL to 16 mg/L OCT. Note that the final concentration of inoculum corresponds to 9 × 10^5^ CFU/mL but was set to 1 × 10^6^ in our experiments. Alternatively, cultures ranging from 1 × 10^7^ to 1 × 10^8^ cells/mL were exposed to a constant OCT concentration of either 0.5, 1 or 2 mg/L. The growth was observed at 420–560 nm over 20 h at 37 °C under shaking in Bioscreen C (Oy Growth Curves Ab Ltd., Helsinki, Finland) plate reader. The lowest concentration of OCT, that resulted in total inhibition of growth was taken as the IC_100%_ which is equivalent to MIC (minimum inhibitory concentration).

### 4.4. Assessment of OCT’s Adsorption on the Bacterial Surface

#### 4.4.1. Calculation of OCT Molecules Bound on Cells as Measured by Optical Density of Bacterial Culture at 420–560 nm after OCT Treatment

IC_100%_ values obtained from the above-described microdilution assay were modelled with a previously published approach [31]. Briefly, the total dose of OCT is decoupled in unbound ([U]) and bound ([B]) molar concentrations. The latter is proportional to bacterial concentration (n_cell_) and hence leads to the number of bound OCT per cell: [B] = N_OCT_ × n_cell_ × 10^3^/N_A_, where N_OCT_ is the number of OCT per single cell and N_A_ = 6.022 × 10^23^/mol is the Avogadro number (see [33] for further details). The factor 10^3^ is needed for conversion from milliliter to liter.

#### 4.4.2. Calculation of OCT Molecules Bound on Cells as Measured by Absorption of OCT at 281 nm after Incubation of OCT with Bacteria

OCT solutions of defined concentrations from 1 to 16 mg/L dissolved in water, which corresponds to 1.8 to 29 µM, were incubated without and with washed mid-log grown cells in PBS buffer of 1 × 10^6^ cells/mL, 1 × 10^7^ cells/mL, 1 × 10^8^ cells/mL and 1 × 10^9^ cells/mL for 5 min at 37 °C. Prior to measurement at 281 nm, samples were harvested at 16,000 g for 5 min and only the supernatant was taken for measurements. The spectra were recorded on JASCO V-630 spectrofluorometer (Biolab, Vienna, Austria) combined with JASCO Spectra Menager software for V-600 series (VWS—771, Biolab, Vienna, Austria). The baseline was corrected by subtraction of buffer samples. Of note, the spectra reordered for 1 × 10^8^ cells/mL or higher exhibited two spectra indicating higher interference coming from cells or cellular components, which were not considered for calculations. The absorbance of OCT at 281 nm is proportional to OCT concentration in the supernatant, i.e., the unbound concentration [U]. Then, the number of OCT (N_OCT_) molecules per single cell was calculated from the definition of unbound fraction [U][OCT] = 1 − N_OCT_ n_cell_ × 10^3^/(N_A_ × [OCT]), where [OCT] is the molar concentration of OCT.

## 5. Conclusions

With regard to the experimental design, even seemingly trivial parameters like the method of cell determination or the buffer system used can significantly influence the interpretation of bacterial susceptibility towards an antiseptic agent. Concerning OCT, no antimicrobial resistance has been reported in clinical isolates to date, although this molecule has been routinely applied in a large field of topical applications for more than 3 decades. Therefore, data from in vitro studies must be critically questioned in terms of their practical relevance in “real world” clinical settings.

## Figures and Tables

**Table 1:** **Characteristic of used *E. coli* strains including MIC values for OCT.** Minimal inhibitory concentration (MIC) was determined for 1 × 10^6^ CFU/mL (colony forming units/mL) where no visible growth was observed at 420–580 nm in the presence of OCT.

*E. coli* Strain	Relevant Genotype	Defect	MIC [mg/L]	Source/Reference
ATCC 25992		wt	1	LGC Standards GmbH, Germany
BW25113 (K-12)	*F- DE(araD-araB)567 lacZ4787(del)::rrnB-3 LAM- rph-1 DE(rhaD-rhaB)568 hsdR514*	LPS O-Antigen	1	[18]
d12	*F-*, *proA23*, *lac-28*, *tsx-81*, *trp-30*, *his-51*, *rpsL173(strR)*, *ampCp-1*	LPS O-Antigen	1	[19,20,21]
d12f2	*F-*, *proA23*, *lac-28*, *tsx-81*, *trp-30*, *his-51*, *rpsL173(strR)*, *rfa-31*, *rfa-1*, *ampCp-1*	LPS O-Antigen Outer core Inner core	1	[19,22,23,24]
MG1655 (K-12)	*F-*, *λ-*, *ilvG*, *rfb-50*, *rph-1*	LPS O-Antigen	1	[25]
BKT12	*F-*, *λ-*, *ilvG*, *rfb-50*, *ΔclsB861*, *ΔclsC788::kan*, *ΔclsA856*, *IN(rrnD-rrnE)1*, *rph-1*	LPS O-Antigen CL	1	[26,27,28,29]
BKT29	*F-*, *λ-*, *ilvG*, *rfb-50*, *ΔaraBAD-714*, *rcsF1386::mini-Tn10cam*, *λ-*, *ΔclsB861*, *ΔymdB787::kan*, *ΔclsC888*, *ΔclsA856*, *lpp-2*, *Δ pgsA792*, *rph-1*	LPS O-Antigen CL, PG	1	[26,27,28,29]

**Table 2:** *E. coli* wildtype cell counts as obtained by indicated methods.

	Literature	Manual Colony Counting	QUANTOM^TM^
**OD_600 nm_**	1	1	1
**CFU/mL**	8 × 10^8^	6.0 ± 1.6 × 108	-
**cells/mL**	8 × 10^8^	-	3.9 ± 0.2 × 10^8^

## Data Availability

Data are available upon request.
